# Secondary Hypogonadism and Effects of Testosterone Replacement Therapy on Cardiovascular Events

**DOI:** 10.1210/clinem/dgaf631

**Published:** 2025-11-18

**Authors:** Vicki Munro, Jocelyn Law, Kara Matheson, Syed Ali Imran

**Affiliations:** Division of Endocrinology, Department of Medicine, Dalhousie University, Halifax, Canada NS B3H 2Y9; Division of Endocrinology, Department of Medicine, Dalhousie University, Halifax, Canada NS B3H 2Y9; Research Methods Unit, Nova Scotia Health, Halifax, Canada NS B3H 3A7; Division of Endocrinology, Department of Medicine, Dalhousie University, Halifax, Canada NS B3H 2Y9

**Keywords:** secondary hypogonadism, HPG axis, cardiovascular safety, pituitary disease

## Abstract

**Context:**

The safety of testosterone replacement therapy (TRT) has generated some controversy during recent years. While untreated hypogonadism leads to diminished sexual characteristics, muscle weakness and osteoporosis, the cardiovascular safety of TRT has been vigorously debated. TRT remains the standard of care for those with secondary hypogonadism (SHG) due to structural pituitary disease, but long-term cardiovascular safety in this population remains unclear.

**Methods:**

We conducted a retrospective cohort study to investigate occurrence of major adverse cardiovascular events (MACE) in male patients with nonfunctioning pituitary adenomas and prolactinomas, with and without SHG. Demographic data, TRT treatment, stroke, myocardial infarction, and mortality data were retrieved from chart review as well as provincial cardiac and stroke registries.

**Results:**

There were 408 patients followed for a median 8.1 years (interquartile range 3.3-14.1); 150 (36.7%) did not have SHG, whereas 214 (52.5%) had SHG adequately treated with TRT and 44 (10.8%) had SHG that was untreated. Multivariable logistic regression analysis demonstrated no significant difference in MACE between groups. MACE outcomes were not impacted by size of adenoma, presence of other pituitary hormonal deficits, or testosterone levels. There was increased risk of mortality in untreated SHG compared to both TRT-treated SHG and those without SHG.

**Conclusion:**

TRT does not appear to increase risk of MACE in those with SHG related to pituitary disorders. Untreated SHG appears to convey increased risk of mortality though these patients were older and more comorbid.

Male hypogonadism is associated with a variety of physical, metabolic, and neurocognitive symptoms including diminished secondary sexual characteristics, muscle weakness, osteoporosis, and sexual dysfunction. Unless otherwise indicated, testosterone replacement therapy (TRT) aimed at normalizing serum testosterone levels is the standard of care (1). However, conflicting studies concerning potential linkage between TRT and adverse cardiovascular events (2-4) led various national agencies to issue statements of possible increased risk of heart attack and stroke (5, 6). Recently, a randomized placebo-controlled trial demonstrated no increased risk of major adverse cardiovascular events (MACE) in hypogonadal men on TRT vs placebo (7). However, those with testosterone <3.5 nmol/L (100 ng/dL) were excluded from the trial, resulting in a baseline median testosterone of 7.87 nmol/L (227 ng/dL), which is much higher than what is typically seen in those with secondary hypogonadism (SHG) from structural pituitary disease. Furthermore, there was no differentiation or investigation into the underlying etiology of hypogonadism and the mean follow-up duration was only 33 months.

The impact of long-term TRT on cardiovascular disease or mortality in those with SHG continues to remain unclear (8). SHG is common in patients with sellar masses, occurring in more than one third of patients with nonfunctioning pituitary adenoma (NFPA) (9), and 29% of patients with pituitary incidentaloma (10). Furthermore, studies have also shown increased standardized mortality rate in patients with hypopituitarism (8), with 1 study showing a higher standardized mortality rate in untreated hypogonadal patients compared with TRT-treated patients (11). To date, no study has evaluated the long-term cardiovascular safety of TRT in this specific hypogonadal population. We sought to address this knowledge gap by assessing MACE in males with SHG.

## Materials and Methods

The Halifax Neuropituitary (HNP) registry, established in November 2005, prospectively collects data on all neuropituitary patients within the province of Nova Scotia, Canada (population ∼1 million). The HNP clinic systematically follows patients with sellar masses based on established protocols for replacing secondary hormonal deficiencies in accordance with specific guidelines described elsewhere (8). Briefly, all patients were assessed 3, 6, and 12 months after initial presentation, followed by annual assessment for 5 years and every 2 years thereafter. At each visit, complete anterior pituitary hormonal assessment (morning serum cortisol, TSH, free thyroxine, LH, FSH, prolactin, testosterone/estradiol, and IGF-1) was conducted. Patients with SHG were diagnosed based on serum total testosterone levels (drawn between 0800 and 0900 h) below the normal reference range (8.00-32.00 nmol/L) and nonelevated FSH and LH, on 2 or more consecutive tests at least 8 weeks apart. Postsurgical patients were assessed 3 months postoperatively with repeat levels before commencing TRT. Choice of TRT (transdermal vs intramuscular) was based on patient preference, cost, and coverage of the medication. TRT in all treated SHG patients was titrated to achieve a morning fasting serum testosterone within mid-normal range; in patients taking depot testosterone, serum testosterone was measured 1 week after the injection. Adherence to the therapy was assessed at each visit by reviewing serum testosterone levels as well as the provincial drug information portal for regular pickup of prescriptions. Those with secondary hypothyroidism were treated with levothyroxine therapy to target a serum free thyroxine level in mid-normal range. Those with secondary hypoadrenalism were treated with hydrocortisone at median daily dose of 15 mg divided into 2 or 3 doses, with frequent monitoring for overreplacement and testing for recovery of hypothalamic-pituitary-adrenal axis, as previously described (12).

We conducted a retrospective cohort review of all males from 2006 to 2023 diagnosed with either NFPA or prolactinomas (PRLoma). Males were divided into 3 cohorts: (1) no SHG, (2) TRT-treated SHG, and (3) untreated SHG. Those with PRLoma who had initial hypogonadism of <1 year’s duration, with recovery after dopamine agonist therapy, were categorized as having no hypogonadism. Similarly, those on treatment for <12 month’s duration were included in the untreated SHG group.

Exclusion criteria included patients with other functional sellar masses (acromegaly and Cushing disease) due to confounding increased baseline cardiovascular risk, primary hypogonadism, and patients with inadequate follow-up data (<1 year of follow-up). Although we did not limit the sample size, our sample size calculation determined that with a type 1 error (α) rate of .05, a total of 398 patients would provide adequate power to detect a >5% difference in the event rate.

The primary outcome was MACE, defined as a composite of nonfatal myocardial infarction (MI), nonfatal stroke, percutaneous coronary revascularization without MI, and cardiac-specific mortality. We cross-referenced patient health card numbers with the Provincial Cardiac Registry and Provincial Stroke Registry to retrieve any admissions for MI and stroke diagnosed after the pituitary (and hypogonadism if applicable) diagnosis. Individual patient charts were reviewed for mortality information (death documents); when cause of death could not be found in the chart, we included these patients as cardiac-specific death. We also reviewed charts for any cardiac revascularization procedures without MI and baseline demographics including age at pituitary diagnosis, type of pituitary adenoma, other hormonal deficiencies present, presence of hypogonadism, and type of TRT. Presence of other cardiovascular risk factors were also recorded if available, including body mass index (BMI), type 2 diabetes, dyslipidemia, smoking history, history of previous cardiovascular disease (CVD), as well statin and antihypertensive medication use. Serum total testosterone was quantified by chemiluminescent microparticle immunoassay on the Architect i2000SR platform (Abbott Diagnostics, Abbott Park, IL, USA). Total cholesterol, high-density lipoprotein cholesterol, and triglycerides (TG) were measured on an Architect c1600 analyzer (Abbott Diagnostics). For samples with TG <4.5 mmol/L, low-density lipoprotein-cholesterol (LDL-C) was calculated using the Friedewald formula LDL-C = total cholesterol − high-density lipoprotein cholesterol − (TG/2.2). Direct LDL-C was measured by homogeneous enzymatic assay on the same platform, when TG threshold was exceeded. Hematocrit (Hct) was determined on a Sysmex XN-9000 analyzer (Sysmex Corporation, Kobe, Japan) using the instrument's automated calculation based on red blood cell count and mean corpuscular volume.

### Statistical Analysis

Descriptive statistics were reported as counts and percentages for categorical variables, means, ­and SD for normally distributed continuous variables, and median and interquartile ranges (IQRs) for nonnormally distributed continuous variables. Patient characteristics were compared between study cohorts using chi-square tests for categorical variables and Kruskal-Wallis test for nonparametric continuous variables. A competing risk analysis was performed on the 2 main cohorts (TRT-treated SHG and no SHG patients) comparing MACE with noncardiac death as competing risk. The cumulative incidence function was compared between cohorts using Gray's method. Univariable analysis using competing risk regression was performed on a priori selected clinical factors, followed by multivariable regression modelling, with SHG group as a fixed effect and baseline characteristics selected according to stepwise selection method. The proportional hazards assumption was tested using Kolmogorov-type supremum test. Subdistribution hazard ratio (SHR) and 95% CIs are reported. Univariable logistic regression followed by multivariable logistic regression was performed to examine the association between the composite outcome MACE events in the primary 2 cohorts: no SHG patients and TRT-treated SHG patients. The SHG group was included in the model as a fixed effect and baseline characteristics were selected according to stepwise selection method. Unadjusted odds ratios (ORs) with 95% CIs were reported. The linearity assumption of continuous variables was verified. Hosmer and Lemeshow Goodness of Fit Test Statistical and area under curve were used to look at model fit. All analyses were performed using SAS STAT 15.1 version 9.4 (SAS Institute, Cary, N.C.). A 2-sided *P* value of <.05 was the threshold for statistical significance unless otherwise stated. The study was approved by the Nova Scotia Health Research Ethics Board.

## Results

Baseline characteristics are summarized in Table 1. A total of 423 male patients with either NFPA or PRLomas were retrieved from the HNP database. Fifteen were excluded because of insufficient follow-up; of the remaining 408 patients, 278 (68.1%) had NFPA and 130 (31.8%) had PRLoma. Patients with NFPA were more likely to have additional hormonal deficiencies (65.8%) compared with patients with PRLoma (39.5%) (*P* < .0001). There were higher rates of secondary hypothyroidism (64.1% vs 39.5%), growth hormone (GH) deficiency (19.9% vs 3.1%), and secondary adrenal insufficiency (37.0% vs 10.9%) in NFPA compared with PRLomas, respectively (*P* < .0001 for all categories).

**Table 1.** Baseline characteristics

	Study group				
	No SHG (N = 150)	SHG—TRT-treated (N = 214)	SHG—untreated (N = 44)	Total (N = 408)	*P*
**Type of adenoma, n (%)**					**.0005** *^a^*
Nonfunctioning	89 (59.3%)	164 (76.6%)	25 (56.8%)	278 (68.1%)	
Prolactinoma	61 (40.7%)	50 (23.4%)	19 (43.2%)	130 (31.9%)	
**Age at diagnosis, y**					**.0087*^b^***
Median (IQR)	54.5 (43.0, 66.0)	58.0 (47.0, 67.0)	63.5 (53.0, 72.5)	57.0 (45.5, 67.0)	
**Adenoma size,** cm					**<.0001*^b^***
Median (IQR)	1.7 (1.2, 2.4)	2.6 (1.9, 3.5)	2.2 (1.5, 3.0)	2.2 (1.4, 3.0)	
**BMI, kg/m^2^**					.1005*^b^*
Median (IQR)	29.2 (25.8, 33.0)	30.0 (27.1, 34.5)	29.1 (25.8, 34.2)	29.7 (26.5, 34.1)	
**Diabetes, n (%)**					.1511*^a^*
No	123 (83.1%)	166 (77.6%)	31 (72.1%)	320 (79.0%)	
Yes	25 (16.9%)	48 (22.4%)	12 (27.9%)	85 (21.0%)	
**Smoking, n (%)**					.7178*^a^*
No	114 (79.7%)	164 (79.6%)	35 (79.5%)	313 (79.6%)	
Yes	29 (20.3%)	42 (20.4%)	9 (20.5%)	80 (20.4%)	
**Dyslipidemia, n (%)**					.5448*^a^*
No	82 (55.4%)	117 (54.9%)	28 (65.1%)	227 (56.2%)	
Yes	66 (44.6%)	96 (45.0%)	15 (34.9%)	177 (43.8%)	
**Hypertension, n (%)**					.5242*^a^*
No	76 (51.0%)	101 (47.2%)	18 (40.9%)	195 (47.9%)	
Yes	73 (49.0%)	113 (52.8%)	26 (59.1%)	212 (52.1%)	
**Preexisting cardiovascular disease, n (%)**					**.0266*^a^***
No	120 (84.5%)	180 (84.9%)	31 (72.1%)	331 (83.4%)	
Yes	22 (15.5%)	32 (15.1%)	12 (27.9%)	66 (16.6%)	
**Other hormonal deficiencies present, n (%)**					**<.0001*^a^***
No	124 (82.7%)	35 (16.4%)	14 (31.8%)	173 (42.5%)	
Yes	26 (17.3%)	178 (83.6%)	30 (68.2%)	234 (57.5%)	
**Secondary hypothyroidism, n (%)**					**<.0001*^a^***
No	120 (82.2%)	36 (17.0%)	20 (45.5%)	176 (43.8%)	
Yes	26 (17.8%)	176 (83.0%)	24 (54.5%)	226 (56.2%)	
**Growth hormone deficiency, n (%)**					**<.0001*^a^***
No	144 (98.6%)	158 (76.7%)	35 (83.3%)	337 (85.5%)	
Yes	2 (1.4%)	48 (23.3%)	7 (16.7%)	57 (14.5%)	
**Secondary hypoadrenalism, n (%)**					**<.0001*^a^***
No	142 (97.3%)	111 (52.4%)	34 (77.3%)	287 (71.4%)	
Yes	4 (2.7%)	101 (47.6%)	10 (22.7%)	115 (28.6%)	
**Recent testosterone level, nmol/L (ref range 8.0-32.0 nmol/L)**					**<.0001*^b^***
Mean (SD)	15.4 (4.76)	16.1 (8.53)	3.1 (2.44)	14.4 (7.92)	

Bolded *P*-values indicate significance <.05.

Abbreviations: BMI, body mass index; IQR, interquartile range; SHG, secondary hypogonadism; TRT, testosterone replacement therapy.

^*a*^Chi-square *P* value.

^*b*^Kruskal-Wallis *P* value.

Median follow-up after the initial diagnosis of pituitary disease was 8.1 years (IQR 3.3-14.1). Approximately two thirds of patients (258) had SHG, and 44 (17.1%) of those were not on TRT. The reasons for being untreated included: (1) declined treatment (26), (2) elevated prostate-specific antigen or prostate cancer (6), (3) noncompliance (4), (4) being observed within 6 months of normalization of prolactin (3), (5) TRT stopped because of side effects (rash to gel) (2), and (6) undetermined by chart review (3). In those treated with TRT, 119 (55.6%) were on intramuscular depot testosterone and 95 (44.4%) on transdermal therapy.

Those with SHG, in comparison to those without, had larger tumors (*P* = .0005) and multiple additional secondary hormonal deficiencies (*P* < .0001). There were no differences in BMI, presence of type 2 diabetes, smoking history, dyslipidemia, or hypertension between groups. There were no differences in statin use between the no SHG (42.7% on statin therapy), TRT-treated SHG (50.5%), and untreated SHG groups (47.7%) (*P* = .630) nor antihypertensive use between the no SHG (45.3% on 1 or more antihypertensive agent), TRT-treated SHG (54.7%), and untreated SHG groups (52.3%) (*P* = .461). The untreated SHG group were older (mean age 61.7 [SD 15.1]) compared with 56.8 (SD 14.1) and 53.7 (SD 16.1) in the TRT-treated and no SHG groups, respectively (*P* = .0087) and had higher rates of preexisting CVD (27.3%) compared with TRT-treated (14.7%) and the no SHG groups (15%) (*P* = .027). There was no significant difference in recent testosterone levels between the no SHG group (median testosterone 14.8 [IQR 11.9, 18.5]) and the TRT-treated SHG group (median testosterone 14.8 [IQR 10.2, 21.4]) (*P* = .75, Ref 8.0-32.0 nmol/L). The untreated SHG group had significantly lower testosterone levels (3.2 nmol/L [IQR 0.6, 5.1], *P* < .001). There were no significant differences in the 3 groups concerning their serum Hct levels with median Hct of 0.4 (Ref 0.42-0.54 L/L), or recent serum LDL levels (no SHG group median LDL 2.4 [IQR 1.7, 3.3], TRT-treated LDL 2.2 [IQR 1.6, 3.0], and untreated SHG LDL 2.3 [IQR 1.7, 3.0] Ref 0.0-3.5 mmol/L; *P* = .18).

### MACE Outcome

**Figure 1.**
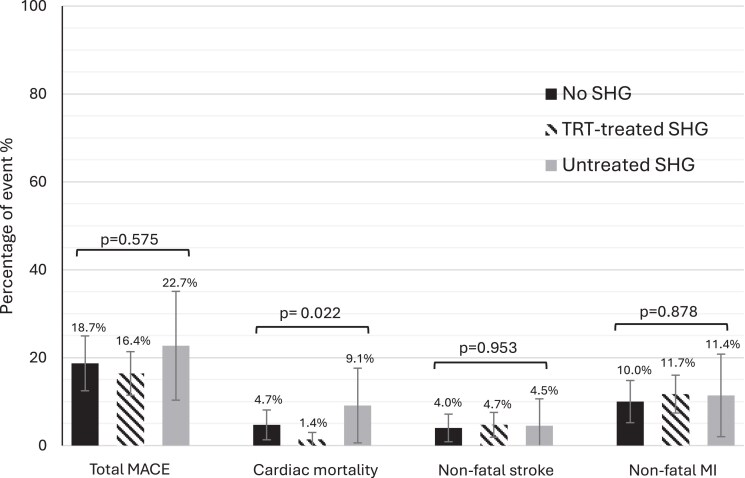
Total MACE, nonfatal stroke events, nonfatal MI events, and cardiac mortality events.

These data are summarized in Fig. 1. A total of 73 (17.9%) patients experienced a MACE. Some patients experienced more than 1 cardiovascular (CV) event during follow-up (ie, initial MI followed by death). There were only 2 patients with revascularization without MI, both in the no SHG group. There was no significant difference between groups in the number of MACE (*P* = .57).

Univariable logistic regression identified significant variables including: age at diagnosis, diabetes, smoking, dyslipidemia, hypertension, preexisting CVD, statin therapy, antihypertensive therapy, LDL, and Hct (Table 2). TRT-treated SHG, type of pituitary adenoma, presence of other hormonal deficiencies, and testosterone levels were not associated with a statistically significant increase in odds of MACE. In the multivariable regression, TRT-treated SHG patients did not demonstrate higher odds of MACE compared with patients with no SHG (OR 0.74 [95% CI, 0.37-1.51] *P* = .413). As expected, odds of MACE were significantly higher in those taking statins (OR 3.79 [95% CI, 1.48-9.72] *P* = .006) or antihypertensive therapy (OR 6.04 [95% CI, 2.11-17.28] *P* = .0008) as well as those with preexisting CVD (OR 5.88 [95% CI, 2.75-12.56] *P* < .0001), whereas BMI was not associated with increased odds of MACE (OR 0.95 [95% CI, 0.89-1.01] *P* = .096).

**Table 2.** Univariable logistic regression of MACE events

	MACE outcome	
	Odds Ratio (95% CI)	*P* value
**Study group—n (%)**		
TRT-treated SHG vs no SHG*^a^*	0.85 (0.49-1.47)	.5563
**Primary diagnosis—n (%)**		
Nonfunctioning adenoma vs prolactinoma*^a^*	1.36 (0.73-2.51)	.3352
**Age at diagnosis**—**(per each 10-y increase in age)**	1.45 (1.19-1.78)	**.0003**
**Diabetes—present vs none*^a^***	3.89 (2.15-7.03)	**<.0001**
**Smoking history—yes vs no*^a^***	2.33 (1.26-4.32)	**.0070**
**Dyslipidemia—present vs none*^a^***	4.39 (2.38-8.14)	**<.0001**
**Hypertension—yes vs no*^a^***	5.70 (2.86-11.37)	**<.0001**
**Preexisting CVD—yes vs no*^a^***	10.85 (5.65-20.84)	**<.0001**
**Other hormonal deficiencies present—yes vs no*^a^***	1.52 (0.86-2.68)	.1490
**Statin therapy—yes vs no*^a^***	9.91 (4.55-21.59)	**<.0001**
**Antihypertensive therapy—yes vs no*^a^***	11.77 (4.92-28.19)	**<.0001**
**Adenoma size**—**per 1-cm increase** (Adenoma on the log scale)	Not linear on log scale 1.04 (0.69, 1.55)	.8510
**BMI—per 1-unit increase**	0.96 (0.92, 1.02)	.1602
**Testosterone level—per-1 nmol/L increase**	0.99 (0.95, 1.03)	.4923
**Recent LDL—per 1-mmol/L increase**	0.41 (0.28, 0.59)	**<.0001**
**Recent Hct—per 0.1-L/L increase**	0.53 (0.33, 0.84)	**.0077**

Bolded *P*-values represent significance <.05.

Abbreviations: BMI, body mass index; CVD, cardiovascular disease; Hct, hematocrit; LDL, low-density lipoprotein; MACE, major adverse cardiovascular event; SHG, secondary hypogonadism.

^*a*^Denotes the reference group.

Within the TRT-treated SHG group, 55.6% were receiving IM injection and 44.4% transdermal gel. There was no significant difference in MACE when comparing treatment formulation, with MACE occurring in 19.33% (23/119) IM users and 12.63% (12/95) of transdermal users (*P* = .1882). The SHR of transdermal gel compared to IM injection was 0.84 (95% CI, 0.42-1.68; *P* = .619).

### Time to First Event Analysis (MACE or Noncardiac Death)

**Figure 2.**
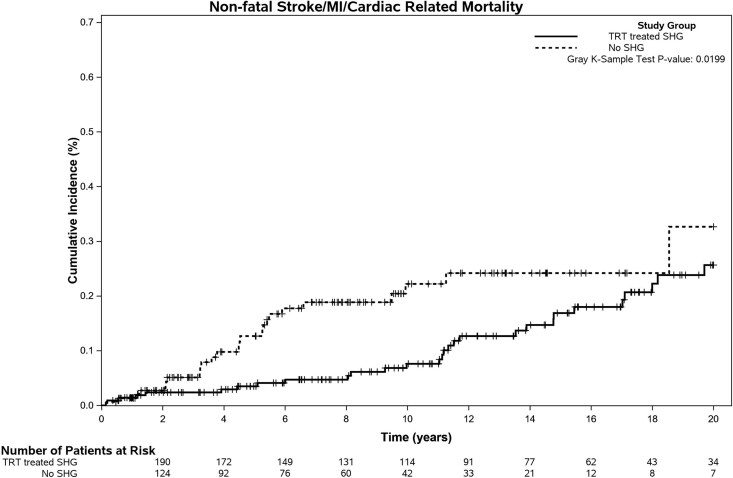
Cumulative incidence curves for the time from diagnosis to MACE by SHG group, adjusting for the competing risk of noncardiac-related death.

**Figure 3.**
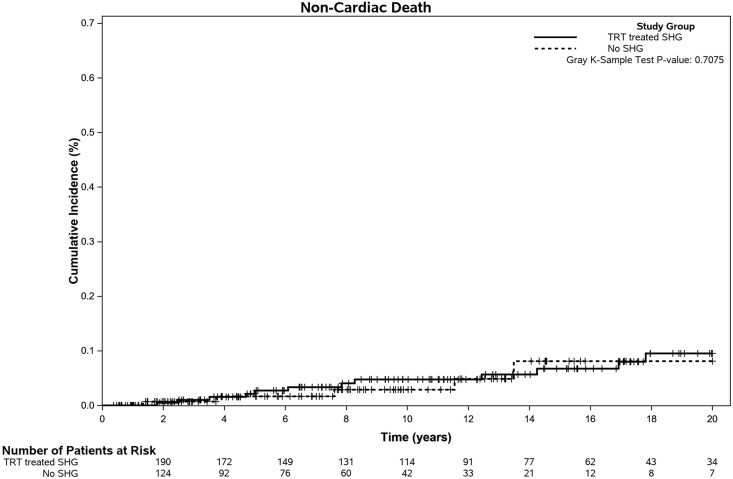
Cumulative incidence curves for the time from diagnosis to noncardiac death by SHG group, adjusting for the competing risk of MACE.

Overall, there were 5 noncardiac deaths in the no SHG group (3.3%), 14 in the TRT-treated SHG group (6.5%), and 9 in the untreated SHG group (20.5%) (*P* = .0013). Figures 2 and 3 illustrate the time to first event of MACE and noncardiac death respectively, in the primary 2 cohorts (no SHG and TRT-treated SHG groups). In univariable competing risk analysis, increased age at diagnosis, presence of diabetes, smoking, dyslipidemia, hypertension, preexisting CVD, statin therapy, antihypertensive therapy, higher LDL, and lower Hct were all associated with worse SHR of development of MACE (Table 3). Only preexisting CVD, increasing age, and lower Hct were associated with worse SHR of noncardiac death. TRT-treated patients with SHG had lower SHR of development of MACE and no significantly increased risk of noncardiac death compared with the no SHG patients.

**Table 3.** Univariable competing risk analysis of MACE and noncardiac death

	Nonfatal stroke/MI/ cardiac-related death		Noncardiac-related death	
	Sub hazard ratio (95% CI)	*P* value	Sub hazard ratio (95% CI)	*P* value
TRT-treated SHG vs no SHG*^a^*	0.56 (0.34-0.91)	**.0204**	1.36 (0.49-3.76)	.5591
Diagnosis—nonfunctioning pituitary adenoma vs prolactinoma*^a^*	1.37 (0.79-2.38)	.2589	8.20 (1.13-59.55)	.0375
Preexisting CVD—yes vs no*^a^*	6.07 (3.59-0.26)	**<.0001**	1.08 (0.36-3.28)	**<.0001**
Diabetes—present vs none*^a^*	2.43 (1.48-3.99)	**.0004**	1.17 (0.41-3.36)	.7662
Smoking history—yes vs no*^a^*	2.37 (1.38-4.08)	**.0018**	1.01 (0.35-2.93)	.9889
Hypertension—yes vs no	4.46 (2.30-8.66)	**<.0001**	1.04 (0.41-2.64)	.9277
Dyslipidemia—yes vs no*^a^*	4.20 (2.36-7.48)	**<.0001**	1.18 (0.47-2.94)	.7248
Other hormonal deficiencies present—yes vs no*^a^*	0.96 (0.57-1.63)	.8782	2.06 (0.67, -6.32)	.2042
Statin use—yes vs no*^a^*	6.93 (3.33-14.43)	**<.0001**	0.47 (0.18-1.28)	.1404
Antihypertensive use—yes vs no*^a^*	7.86 (3.38-18.23)	**<.0001**	1.15 (0.44-3.03)	.7766
Age per 10-y increase	Not linear on logarithmic scale 2.75 (1.92-3.93) **< .0001**		2.25 (1.64-3.09)	**<.0001**
Age at diagnosis (y)				
Age <40*^a^*	Reference		0	<.0001
40 to <50	1.68 (0.67-4.23)	.2717	0	<.0001
50 to <60	1.61 (0.66-3.94)	.2965	Reference	—
60 to <70	2.36 (1.02-5.46)	.0456	2.18 (0.59, 7.98)	.2410
≥70	7.11 (3.13-16.15)	**<.0001**	4.80 (1.31-17.52)	.0176
Adenoma size Per 1-cm increase	0.93 (0.85-1.01)	.0872	0.96 (0.86-1.08)	.5167
BMI Per 1-unit increase	0.99 (0.93-1.05)	.6171	Not linear on logarithmic scale	
Recent testosterone level Per 1-nmol/L increase	0.99 (0.97-1.03)	.7584	1.00 (0.94-1.07)	.9831
Recent LDL Per 1-mmol/L increase	0.50 (0.37-0.67)	**<.0001**	0.96 (0.49-1.87)	.5049
Recent Hct Per 0.1-L/L increase	Not linear on logarithmic scale 0.43 (0.23-0.81) **.0326**		0.30 (0.17-0.65)	.0013

Bolded *P*-values represent significance <.05.

Abbreviations: BMI, body mass index; CVD, cardiovascular disease; Hct, hematocrit; LDL, low-density lipoprotein; MACE, major adverse cardiovascular event; SHG, secondary hypogonadism; TRT, testosterone replacement therapy.

^*a*^Denotes the reference group.

In the multivariable analysis, after adjusting for age, preexisting CVD, statin and antihypertensive medication use, TRT-treated patients with SHG had significantly lower risk of developing MACE, with SHR of 0.45 (95% CI, 0.24-0.85; *P* = .013). The other variables included in multivariable analysis continued to contribute significantly increased risk of MACE as shown in Table 4.

**Table 4.** Multivariable competing risk analysis of MACE and noncardiac death

	*P* value	Sub hazard ratio	95% sub hazard ratio confidence limits	
TRT-treated SHG vs No SHG*^a^*	**.0131**	0.450	0.24	0.85
Age—global *P* value	.0572			
Age 40 to <50 vs age <40	.1913	1.89	0.73	4.95
Age 50 to <60 vs age <40	.4449	1.49	0.54	4.12
Age 60 to <70 vs age <40	.1379	1.994	0.801	4.96
Age 70+ vs age <40	**.0048**	4.383	1.57	12.236
Preexisting CVD—yes vs no*^a^*	**.0008**	2.84	1.55	5.22
Statin therapy—yes vs no*^a^*	**.0014**	4.46	1.78	11.15
Antihypertensive therapy—yes vs no*^a^*	**.0055**	3.94	1.49	10.37

Bolded *P*-values represent significance <.05.

Abbreviations: CVD, cardiovascular disease; MACE, major adverse cardiovascular event; SHG, secondary hypogonadism; TRT, testosterone replacement therapy.

^*a*^Denotes the reference group.

## Discussion

CV safety of TRT in hypogonadal men has been vigorously debated. The effects of testosterone on the heart are mediated through androgen receptors that belong to the steroid receptor superfamily. There is ample evidence to suggest that testosterone exerts a direct effect on myocardium and blood vessels, and androgen receptors are expressed in the myocardium (13). Testosterone also modulates cardiac adaptive response to injury (14), improves cardiac cell mitochondrial function (15), cardiac function, and quality of life in individuals with congestive heart failure (16). However, supraphysiologic testosterone levels are considered detrimental and may lead to cardiac hypertrophy (17). Furthermore, low testosterone has also been associated with a prolonged corrected QT interval (18), which may increase risk of cardiac arrhythmias. Taken together, these findings suggest that TRT aimed at maintaining a serum testosterone within physiological range should be beneficial to the heart.

Despite the physiological rationale, clinical studies have shown somewhat contradictory results. For instance, an initial randomized placebo-controlled trial designed to evaluate muscle mass and strength in older men with mobility limitations was discontinued early because it showed numerically higher CV-related events in the testosterone-treated group (23 vs 5) (2). A subsequent retrospective cohort study of individuals undergoing coronary angiogram reported that use of TRT was associated with an increased risk of adverse CV outcomes among veterans (4). Conversely, other studies have shown reduction in MI, stroke, and all-cause mortality with TRT aimed at normalizing testosterone levels (19, 20), and a meta-analysis also showed no evidence to support an increased CV risk in TRT individuals (21). Indeed, the large multicenter placebo-controlled TRAVERSE trial enrolling men with high risk of CVD did not demonstrate increased incidence of MACE with TRT (7).

The variability in these outcomes may in part be due to differences in methodology, study population, type of TRT, and other unaccounted factors. For instance, some studies used health system databases (3, 4) and were unable to document normalization of serum testosterone levels after initiation of TRT. Data in the SHG population are especially lacking, with hypopituitarism guidelines commenting that CV safety of TRT in this population remains unclear (8). To our knowledge, this is the first study assessing the well-defined population of SHG patients with known pituitary abnormalities who are definitively recommended TRT.

Another issue is the variability in duration of TRT in different studies. For instance, a recent meta-analysis reviewing these data included studies mostly using TRT for only 6 to 12 months’ duration (22). The TRAVERSE trial (7) followed patients for a mean of 33 months; however, those with severe hypogonadism and a testosterone <3.5 nmol/L were excluded from participation, which encompasses many patients with SHG due to pituitary conditions (as highlighted by the median testosterone of 3.1 nmol/L in our untreated SHG group). A major strength of our study is the prolonged follow-up with median of 8.1 years’ duration. Our data support CV safety with long-term TRT use.

Although our study showed that untreated SHG patients had increased risk of both cardiac and noncardiac mortality, it was underpowered to assess differences in this small sample size. Reverse causality also cannot be excluded. Of the untreated SHG patients in our cohort, 6 did not receive TRT due to elevated prostate-specific antigen or prostate cancer. Patients with advanced prostate cancer treated with androgen deprivation therapy appear to have worsened body composition and metabolic profile and also show association with CV morbidity. However, those requiring androgen deprivation therapy often have poorer health status and may have elevated CVD related to selection bias (23). The same may hold true in other untreated SHG patients. In our cohort, these patients were older and had higher baseline rates of preexisting CVD. Those who declined TRT within the untreated group often did so due to self-perceived poor health (often related to cancer or dementia); frailty is also not captured in these parameters.

Our data showed that patients with NFPA had an increased SHR of 8.20 compared to PRLoma of noncardiac death. Although patients with NFPA had higher rates of additional hormonal deficits, they were placed on appropriate hormonal replacement therapies that were consistently monitored for adequacy. The role of hypopituitarism per se is still unclear and previous meta-analyses have demonstrated increased mortality in hypopituitary patients despite taking replacement therapy (24, 25). A higher mortality in NFPA compared with the general population has been previously reported, particularly in individuals who are older at diagnosis (26). We previously reported that the mean age at diagnosis in males with NFPA was 52 years as opposed to 37 years in PRLoma (9), which may partly explain the higher mortality in this group.

There were several limitations to our study. Our initial sample size calculation suggested that our population of pituitary patients could sufficiently detect a >5% difference in MACE outcomes but may be underpowered for smaller differences. Though thorough chart review was performed on each patient, some deaths occurring outside the hospital may not have been captured in the medical records and could have been missed. Untreated patients with SHG were more likely to have elevated PSA levels and had PRLoma. Additionally, despite the established safety of dopamine agonist therapy in pituitary population, its possible effect on cardiovascular outcomes could not be fully excluded. We limited our population to those with NFPA and PRLoma; therefore, our results on TRT may not be applicable to those with other pituitary abnormalities causing hypopituitarism, such as nonpituitary sellar masses, GH, and adrenocorticotrophic secreting adenomas, or other causes of hypopituitarism. Given this was a retrospective review, patients were not randomized in receiving treatment or in what formulation of TRT they received.

## Conclusion

To our knowledge, this study represents the first demonstration of CV safety of TRT in patients with SHG with pituitary disease. Despite having higher rates of other hormonal deficiencies, TRT-treated patients with SHG had no increased MACE risk compared with the no-SHG patients and untreated-SHG patients. In keeping with previous results, untreated SHG was associated with higher mortality; however, these patients often had more severe comorbidities, leading to a decision to decline therapy. Our study provides further evidence of CV safety of TRT and may help reassure any patients who decline therapy due to concerns of adverse CV effects.

## Data Availability

Some or all datasets generated during and/or analyzed during the current study are not publicly available but are available from the corresponding author on reasonable request.

## Abbreviations

BMI: body mass index
CV: cardiovascular
CVD: cardiovascular disease
Hct: hematocrit
HNP: Halifax Neuropituitary registry
IQR: interquartile range
LDL: low-density lipoprotein
MACE: major adverse cardiovascular event
MI: myocardial infarction
NFPA: nonfunctioning pituitary adenoma
PRLoma: prolactinoma
SHG: secondary hypogonadism
SHR: subdistribution hazard ratio
TG: triglyceride
TRT: testosterone replacement therapy
